# Comparative efficacy of non- pharmacological interventions on sleep quality in patients with multiple sclerosis: a systematic review and network meta-analysis

**DOI:** 10.7717/peerj.20900

**Published:** 2026-03-16

**Authors:** Yanping Liu, Xiaoli Zhao, Congying Luo, Ai Chen, Xiaopeng Zeng

**Affiliations:** 1 Department of Neurology, The First Affiliated Hospital of Chongqing Medical University, Chongqing, China; 2 Department of Nursing, The First Affiliated Hospital of Chongqing Medical University, Chongqing, China

**Keywords:** Multiple sclerosis, Non-pharmacological interventions, Sleep quality, Network meta-analysis, Randomized trial

## Abstract

**Background:**

This study aimed to evaluate the effectiveness of non-pharmacological interventions for improving sleep quality in patients with multiple sclerosis (MS) using a systematic review and network meta-analysis.

**Method:**

Randomized controlled trials examining the effects of non-pharmacological interventions on sleep quality in patients with MS were retrieved from PubMed, EMBASE, Cochrane Library, Web of Science, CINAHL, CNKI, Wanfang Database, and VIP Database. The search period spanned from database inception to October 31, 2025. We performed a network meta-analysis using RevMan and Stata software.

**Results:**

We included 35 studies involving 2,804 participants and 20 distinct intervention types. The most frequently investigated interventions were exercise-based therapies and cognitive-behavioral approaches. Based on cumulative ranking probabilities, assessed using the surface under the cumulative ranking curve (SUCRA), occupational therapy-based sleep interventions were the most effective for improving subjective sleep quality (SUCRA = 94.2%), followed by mindfulness intervention (SUCRA = 85.9%) and sleep hygiene education (SUCRA = 78.6%). For reducing insomnia severity, effleurage massage ranked highest (SUCRA = 91.9%), followed by cognitive behavioral therapy (SUCRA = 80.1%) and reflexology (SUCRA = 77.0%).

**Conclusions:**

Occupational therapy-based sleep interventions and effleurage massage appear to be the most effective non-pharmacological strategies for improving sleep quality in patients with MS. However, further high-quality randomized controlled trials are needed to confirm these findings and strengthen the evidence base.

## Introduction

Multiple sclerosis (MS) is a chronic autoimmune-mediated disorder of the central nervous system, characterized by demyelination, neuroinflammation, and axonal damage. MS affects approximately 2.8 million individuals worldwide, with a global prevalence of 35.9 per 100,000 people (Walton et al., 2020). The etiology of MS is multifactorial, involving complex interactions among genetic susceptibility, environmental exposures, infectious agents, lifestyle factors, and immune dysregulation (Dobson & Giovannoni, 2018; Tarlinton et al., 2020). Clinically, MS presents in several phenotypic forms, including clinically isolated syndrome, relapsing-remitting MS, secondary progressive MS, and primary progressive MS (Jacques, 2015). These disease courses are associated with heterogeneous symptoms such as fatigue, motor impairment, sensory impairment, and cognitive decline (Ward & Goldman, 2022). Sleep disorders are among the most common complications experienced by individuals with MS. Approximately 60% of adults with MS report clinically significant sleep disturbances (Sakkas et al., 2019). Common sleep disorders in this population include insomnia, obstructive sleep apnea, restless legs syndrome, and excessive daytime sleepiness. These disturbances exacerbate neurological symptoms and impair health-related quality of life (Golabi et al., 2024).

The etiology of sleep dysfunction in MS is multifactorial. Primary mechanisms relate directly to MS pathophysiology, including brainstem and hypothalamic lesions that disrupt sleep-wake regulatory pathways (Majde & Krueger, 2005; Ditmer et al., 2021). Secondary contributors encompass neuropathic pain, spasticity, nocturia, depression, and medication-related side effects, as well as behavioral and psychosocial factors such as reduced physical activity and stress (Nagaraj et al., 2013; Solaro, Trabucco & Messmer Uccelli, 2013; Hughes, Dunn & Chaffee, 2018; Dagnew et al., 2024; Aoun, Attarian & Graham, 2024; Golabi et al., 2024). This multifactorial pathophysiology highlights the need for integrated and individualized approaches to the management of sleep disorders in MS.

Current management strategies for sleep disorders in patients with MS are broadly categorized into pharmacological and non-pharmacological interventions. Pharmacological treatments commonly include antidepressants, anxiolytics, antihistamines, and benzodiazepines. However, these drugs are frequently associated with undesirable side effects and certain psychological and physiological risks, including depression, cognitive impairment, daytime sedation, tolerance, and dependence (Zhang et al., 2022). Conversely, non-pharmacological interventions, including cognitive behavioral therapy for insomnia (CBT-I), mindfulness meditation, physical exercise, sleep hygiene education, and occupational therapy, have gained attention because of their favorable safety profiles and potential to improve sleep, comorbid fatigue, mood, and functional outcomes in patients with MS (Lorenz et al., 2020; Tollár et al., 2020; Drerup et al., 2021).

Recent systematic reviews and meta-analyses have demonstrated that non-pharmacological interventions are effective in improving sleep quality among patients with MS (Turkowitch et al., 2024; Yu, Foidel & Tomlin, 2025). Notably, a comprehensive systematic review with meta-analysis (Ozdogar & Kalron, 2025). Synthesized evidence from nine randomized controlled trials reported a significant overall benefit of non-pharmacological interventions on sleep quality in patients with MS. However, the authors explicitly noted critical limitations within the existing literature, including considerable heterogeneity across studies, variability in intervention protocols (type, duration, and delivery), and a lack of direct comparisons between different active non-pharmacological modalities. Importantly, while a conventional meta-analysis can estimate the overall effect of an intervention class *versus* control, it cannot answer the clinically pressing question of which specific non-pharmacological intervention is the most effective.

Given the diversity of available non-pharmacological interventions and the clinical imperative to identify optimally effective approaches, there is a compelling rationale for conducting a network meta-analysis (NMA). This approach allows for the evaluation of comparative efficacy and the generation of intervention rankings, thereby informing evidence-based decision-making for patients and clinicians. Therefore, this study aims to perform a systematic review and NMA to evaluate and rank the effectiveness of non-pharmacological interventions for improving sleep quality in patients with MS, with the goal of providing robust, evidence-based guidance for clinical practice and future research.

## Methods

This systematic review was registered in the International Prospective Register of Systematic Reviews (PROSPERO; registration number CRD42024555615). The review was conducted in accordance with the COCHRANE system review manual (Cumpston et al., 2019) and the Preferred Reporting Items for Systematic Reviews and Meta-Analyses (PRISMA) statement (Hutton et al., 2015) and considered the recent methodological advancements identified in the ongoing update to the PRISMA-NMA guideline (Veroniki et al., 2025).

### Search strategy and study selection

We comprehensively searched databases, including PubMed, Embase, Cochrane Library, Web of Science, the Cumulative Index to Nursing and Allied Health Literature, China National Knowledge Infrastructure, Wanfang, and VIP information databases to identify studies examining the effects of non-pharmacological interventions on sleep quality in patients with MS. The search covered the period from database inception to October 31, 2025. Search strategies were adjusted according to different databases by combining keywords with free words. The search was performed using keywords such as “multiple sclerosis,” “sleep disorders,” and “non-pharmacological interventions”. The manuscript title and abstract were screened based on eligibility criteria, and at least two independent reviewers conducted full-text screening. Disagreements were resolved through discussion by a third reviewer.

### Eligibility and exclusion criteria

The Population, Intervention, Comparator, Outcomes, Study (PICOS) framework guided the definition of eligibility criteria. (1) Population: we included studies that enrolled adults (≥18, years) diagnosed with MS. We did not restrict inclusion to patients with a pre-existing sleep disorder diagnosis or clinically significant sleep complaints at baseline. This approach was adopted to allow for a comprehensive evaluation of the potential benefits of non-pharmacological interventions across the MS population, including those with established sleep disturbances and those who might benefit from preventive or health-promoting strategies. All participants in the intervention and control groups were required to be receiving medications for MS. (2) Interventions: eligible interventions included any non-pharmacological interventions, without restrictions on frequency, duration, style, format, intensity, or setting. (3) Comparators: eligible comparators included sham control, waitlist control, or conventional treatment (including routine care, supportive guidance such as health education and sleep hygiene advice), physiotherapy, or alternative non-pharmacological interventions distinct from those administered in the experimental group. Original trials that compared only different forms of the same intervention were excluded. (4) Outcomes: primary efficacy outcomes were defined as pre–post changes in the Pittsburgh Sleep Quality Index (PSQI) and the Insomnia Severity Index (ISI). This focus on patient-reported outcomes was selected because most existing randomized trials in this field have utilized these validated subjective measures, whereas studies that employed objective sleep measures (actigraphy and polysomnography) are currently scarce. (5) Study design: only randomized controlled trials were included, with no regional or publication restrictions.

We excluded studies under the following conditions: (1) Full texts were unavailable, or data could not be extracted; (2) Studies that were only registered trial protocols without subsequent clinical trial results; (3) Publications that were in the form of conference abstracts or reviews; (4) Duplicate publications. In cases of multiple reports stemming from the same population, we included only the latest or most informative version to avoid data overlap; (5) Studies published in any language other than English or Chinese were excluded.

### Data extraction

Two authors (LYP and CA) independently extracted data from the included studies. Data extraction and quality assessment results were cross-checked, and any discrepancies were resolved through consensus with a third author (ZXP). Extracted data, including the first author, country, year, sample, baseline characteristics (age and sex), Expanded Disability Status Scale (EDSS), disease duration, disease stage, intervention details (type, frequency, strength, and duration), and sleep-related outcomes (sleep evaluation tools, baselines, the average values after intervention, and the standard deviation of sleep assessment), were entered into the Excel electronic table. Mean values and standard deviation at baseline and the two time points were first extracted directly from original articles whenever available; if unavailable, they were calculated using validated computational methods. Studies for which the required statistics could not be obtained through either approach were excluded from the outcome analysis. Herein, the sham control, conventional treatment, and waitlist control were collectively categorized as the control group.

### Quality evaluation

Risk of bias for each included randomized controlled trial was independently assessed by two investigators using the Cochrane Risk of Bias Assessment Tool for Randomized Trials (RoB 2.0). This assessment framework comprises five core domains: (1) Bias arising from the randomization process; (2) Bias due to deviations from intended interventions; (3) Bias resulting from missing outcome data; (4) Bias in outcome measurement; (5) Bias in the selection of reported outcomes. Each domain was rated as “low risk,” “some concern,” or “high risk” based on the information reported in the original study. The overall risk of bias was defined as follows: low (all five domains rated low risk), some concern (at least one domain rated some concern), and high (one or more domains rated high risk) (Sterne et al., 2019). Disagreements between them were resolved through consensus discussion; if a resolution could not be reached, a third independent reviewer made the final decision.

### Statistical analysis

A traditional meta-analysis was conducted to directly compare the efficacy of different non-pharmacological interventions (including cognitive behavioral therapy, mindfulness intervention, and aerobic exercise) on primary sleep-related outcome measures (PSQI score, ISI score) using RevMan software (version 5.3). For continuous data, we opted to use the standardized mean difference (SMD) with its 95% confidence interval (CI) to express the effect size.

Heterogeneity among the included studies was assessed using the I^2^ statistic. Given the anticipated clinical and methodological heterogeneity across interventions and study populations, we primarily employed a random-effects model for all meta-analyses (pairwise and network), as it accounts for heterogeneity by assuming that the true treatment effects vary across studies. A fixed-effects model was considered only when heterogeneity was negligible (I^2^ ≤ 50%), and studies were deemed sufficiently homogeneous. To further investigate potential sources of heterogeneity in the NMA, we performed inconsistency tests (global and local, *via* node-splitting) to ensure the coherence of direct and indirect evidence within the network. When more than 10 studies were included, funnel plots were generated, and publication bias was evaluated using Egger’s test (Egger et al., 1997) implemented in Stata software (Version 18.0).

Subsequently, NMA was performed using Stata software (Version 18.0) to comprehensively compare the efficacy of various interventions in improving sleep quality among patients with MS and rank the interventions. Network plots were utilized to depict the comparison network between different interventions and the control group. In the plot, nodes represent interventions, with their sizes proportional to the sample size; lines represent studies with direct comparisons, with the thickness of lines proportional to the number of comparative studies.

Before data synthesis, the consistency assumption of the network analysis was examined. When closed loops existed in the network, node splitting analysis was conducted to assess the consistency between direct and indirect comparisons. *P* >  0.05 indicated no significant inconsistency, and a consistency model was subsequently adopted for analysis. A random-effects model was applied for the NMA in this study, and the results were presented as SMD with its 95% CI.

The surface under the cumulative ranking curve (SUCRA) was utilized to assess the probability of each intervention being the most effective. SUCRA values range from 0% to 100%. A higher SUCRA value indicates a higher ranking of the intervention based on efficacy.

## Results

### Literature search results

**Figure 1:**
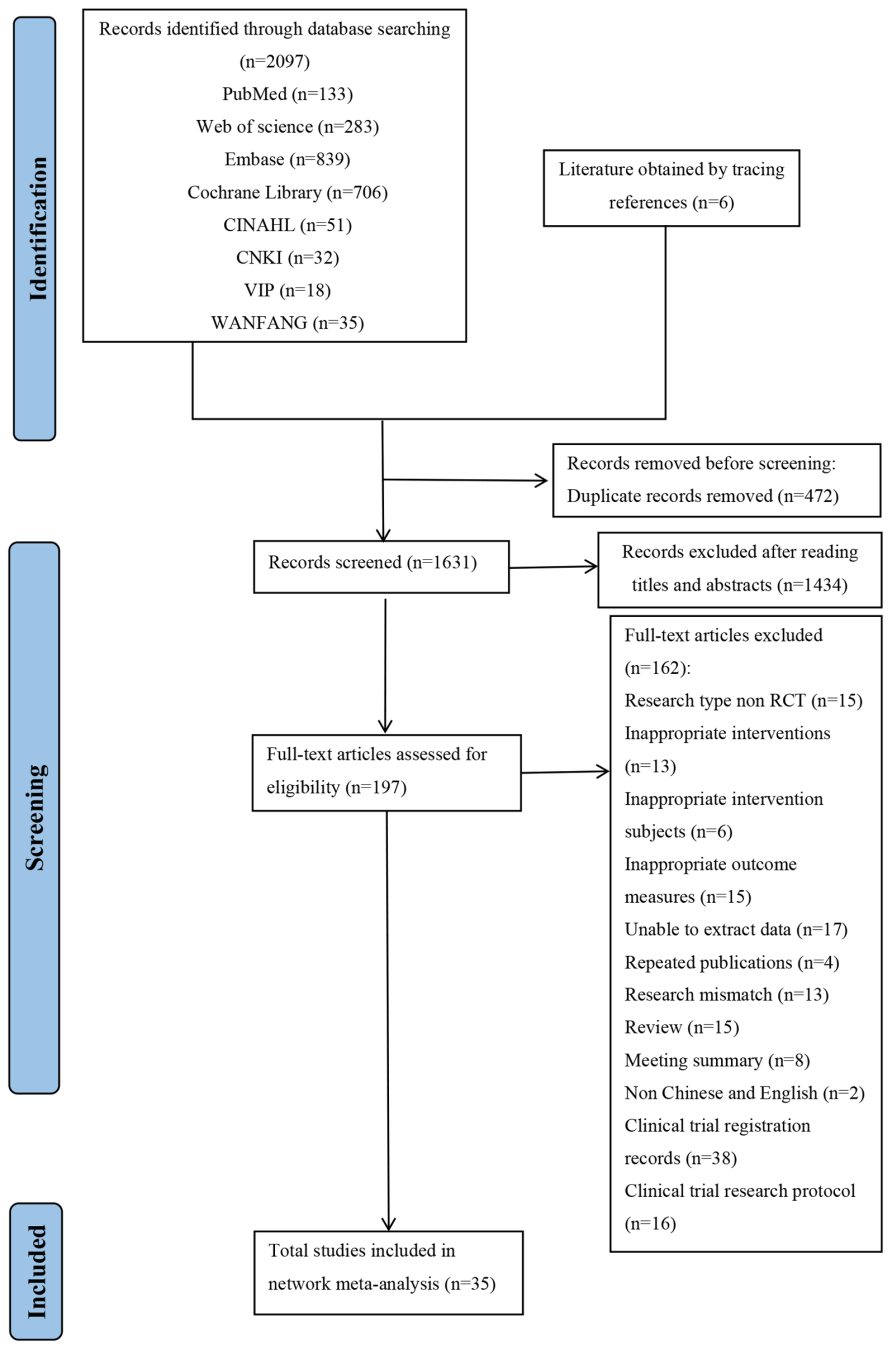
Flow diagram for search and selection of the included studies.

We retrieved 2,103 articles at the initial database search. Additional six studies were retrieved through backward citation tracking of the reference list from included articles and relevant systematic reviews. A total of 1,906 studies were excluded based on duplicate records, irrelevant titles, or abstracts that did not meet the eligibility criteria. The remaining 197 articles underwent full-text screening, of which 35 randomized controlled trials met the inclusion criteria and were included in the systematic review and NMA. Figure 1 depicts the detailed screening process.

### Study characteristics

This study included 35 randomized controlled trials. Table 1 presents their basic characteristics. These studies were published between 2014 and 2025, covering multiple countries, including China, the United States, Iran, Turkey, Canada, Australia, Austria, Italy, Croatia, and France, with a total of 2,804 participants. All participants were clinically diagnosed with MS, with most studies reporting a mean age ranging from 30 to 55 years. Most included patients had EDSS scores between 1.0 and 6.5, indicating mild to moderate disability. The included studies covered various MS subtypes, including relapsing-remitting, secondary progressive, and primary progressive forms. The disease duration ranged from several months to more than 20 years, and female participants accounted for the majority.

This NMA evaluated 20 types of non-pharmacological interventions. Details of the interventions were as follows: (1) Aerobic exercise (three studies), (2) Resistance training (four studies), (3) Mind-body exercise (seven studies), (4) Aerobic exercise + resistance training (one study), (5) Individualized exercise (four studies), (6) Effleurage (three studies), (7) Cognitive behavioral therapy (six studies), (8) Tele-cognitive behavioral therapy (two studies), (9) Mindfulness intervention (two studies), (10) Continuous positive airway pressure (two studies), (11) Behavioral therapy (three studies), (12) Fatigue: take control (one study), (13) Occupational therapy (OT) -based sleep interventions (one study), (14) Bright light therapy (one study), (15) Reflexology (three studies), (16) Aromatherapy (one study), (17) Music therapy (one study), (18) Sleep hygiene (two studies), (19) Acceptance and commitment therapy (one study), and (20) Control group (29 studies). Among these, exercise interventions as a broad category. Exercise-based interventions constituted the most frequently studied non-pharmacological approach. Among the 35 included studies, 12 evaluated exercise, including the preceding five non-pharmacological interventions. These interventions duration ranged from 2 weeks to 6 months, with a frequency of mostly 2–3 times per week and a single session lasting 20–60 min. Among all eligible studies, six studies were three-arm studies, and the remaining 29 articles were two-arm studies.

The primary outcome measures focused on the assessment of sleep quality. PSQI was adopted in most studies, and some studies simultaneously utilized the ISI as an evaluation tool. Overall, the included studies exhibited significant clinical and methodological heterogeneity regarding intervention type, implementation form, sample characteristics, and treatment course, providing a relatively comprehensive evidence base for the systematic evaluation of the effects of different non-pharmacological interventions on sleep quality in patients with multiple sclerosis.

### Quality assessment of the included studies

**Figure 2:**
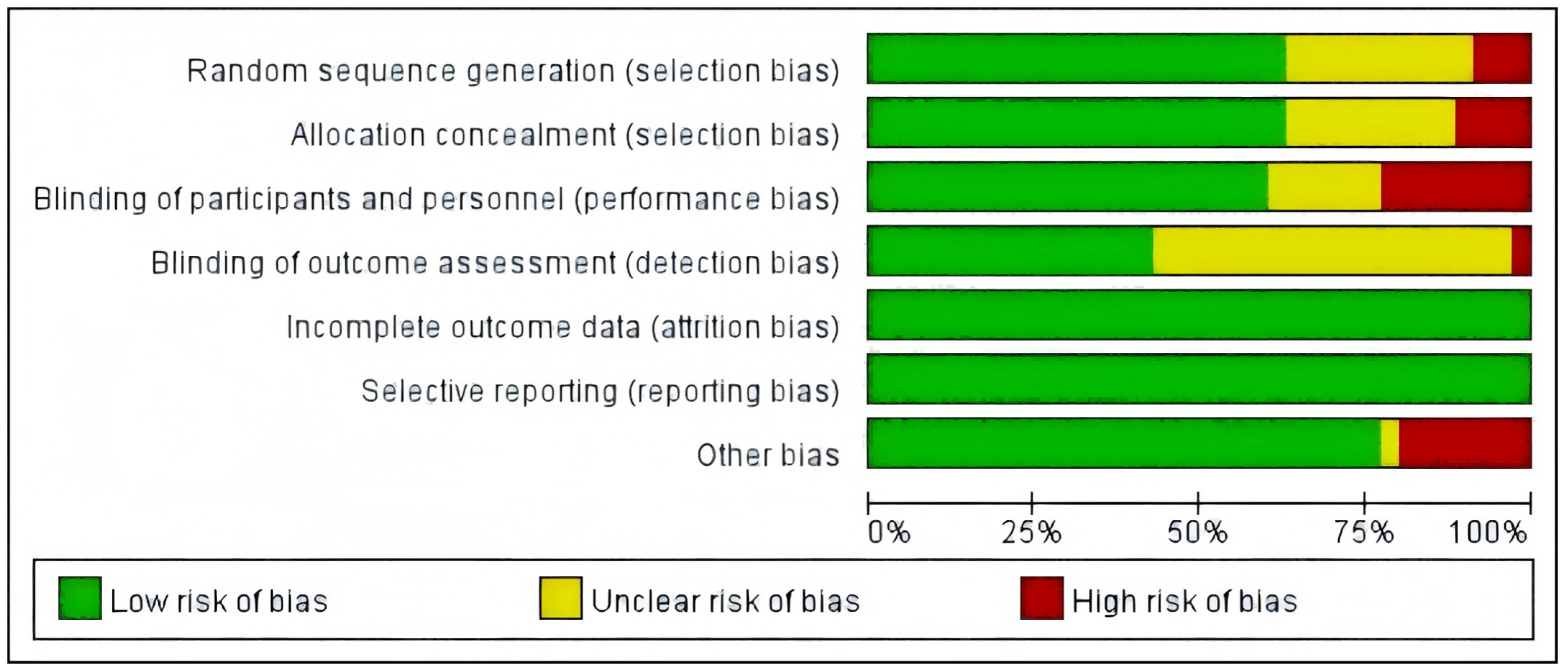
Quality assessment of the eligible studies.

Among the 35 studies analyzed, the assessment of bias risk revealed the following distribution: For random sequence generation, 25 studies were classified as low risk, three were classified as high risk, and seven were classified as unclear because of inadequate reporting. A parallel risk profile was observed in allocation concealment. Regarding blinding implementation, participant and personnel blinding was considered as low risk in 20 studies, was considered as high risk in eight studies, and was unclear in seven studies. Regarding outcome assessor blinding, 16 studies demonstrated low risk, 16 were categorized as unclear, and three were considered as high risk. No missing outcome data were reported across all studies, resulting in a unanimous low-risk rating for outcome data completeness. Similarly, all studies were considered low risk for selective outcome reporting. Additionally, eight studies exhibited high risk concerning other potential bias sources, suggesting possible flaws in methodology or study conduct, whereas one study was considered unclear in this domain. Figure 2 illustrates a comprehensive summary of the quality appraisal.

**Table 1:** Characteristics of included trails network meta-analysis.

Author(year) (country)	Sample (I/C)	Age (I/C)	Period of illness (MS type)	EDSS	Duration of disease (years)	Gender (M/F)	Intervention	Details of interventions (I)	Measured outcomes
Siengsukon et al. (2016) (America)	12/10	I:48.9 ± 13.6 C:50.9 ± 12.2	RRMS/SPMS I:10/2 C:9/1	1.0–6.0	I:10.8 ± 8.4 C:9.0 ± 5.6	3/19	I: AE C: RT	three times a week, for a total of 36 exercise sessions, lasted 12 weeks	PSQI
Voggenberger et al. (2022) (Austria)	13/13	I:42.5 ± 13.4 C:43.4 ± 8.9	NA	≤6.5	I:10.3 ± 11.2 C:14.8 ± 7.1	4/22	I: BLT C: Control	30 min each morning for two weeks, followed by a two-week washout period, lasted 4 weeks	PSQI
Guarnaccia et al. (2023) (America)	24/26	I:50.8 ± 10.1 C:50.9 ± 10.8	NA	≤6.0	NA	9/41	I: Mindfulness intervention C: SH	I:two-hour weekly sessions, ten times, lasted 10 weeks C: one hour sleep hygiene session	PSQI, ISI
Bahçecioğlu Turan, Özer & Arıkan (2024) (Turkey)	30/30	NA	NA	NA	NA	NA	I: ME C: Control	Reiki performance for a total of 3 weeks, 7 days and 20–30 min per session	PSQI
Akbarfahimi et al. (2020) (Iran)	10/10	I:37.50 ± 8.89 C:39.70 ± 7.90	NA	1.0–4.5	NA	4/16	I: OT sleep interventions C: Control	2–3 sessions per week lasting 30–45 min,lasted 8 weeks	PSQI
Naderi et al. (2024) (Iran)	32/34	I: 34.5 ± 10.4 C: 37.0 ± 12.9	RRMS/PPMS/SPMS I:26/1/5 C: 25/3/6	1.0–4.5	I: 9.7 ± 5.2 C: 11.7 ± 6.9	17/49	I: Effleurage C: Control	50 min massage per week, two sessions per week, lasted 6 weeks	PSQI
Zinat et al. (2019) (Iran)	30/30	NA	NA	1.0–4.5	NA	25/35	I: Effleurage C: Control	two sessions of 20–25 min two times a week, lasted 10 weeks	PSQI
Sajadi, Davodabady & Ebrahimi-Monfared (2020) (Iran)	33/30	NA	NA	1.0–4.0	NA	4/59	I: Reflexology C: Control	twice a week and each session lasted about 30–40 min, lasted 4 weeks	PSQI
Pilutti et al. (2014) (America)	41/41	I:48.4 ± 9.1 C:49.5 ± 9.2	RRMS/SPMS/PPMS I: 31/8/2 C:34/2/5	2.5 ± 1.4	I:10.6 ± 7.1 C:13.0 ± 9.1	20/62	I: BT C: Control	seven times during the first 2 months, four times during the second 2 months, and twice during the final 2 months, lasted 6 months	PSQI
Al-Sharman et al. (2019) (America)	17/13	I:38.7 ± 13 C:31.9 ± 10	NA	2.0–5.0	I:9.6 ± 8.49 C:5.43 ± 4.2	7/23	I:AE C: ME	I: 50–60 min aerobic exercise, three times per week ,lasted 6 weeks C: 50–60 min home non-aerobic exercise, three times per week, lasted 6 weeks	PSQI, ISI
Abbasi, Alimohammadi & Pahlavanzadeh (2016) (Iran)	33/33	I:35.3 ± 5.3 C:32.2 ± 8.9	NA	1.0–4.0	Median (IQR) I:5.6(5.8) C:6.1 (6.5)	0/66	I: CBT C: Control	eight 90-minute sessions and one session per week, lasted 8 weeks	PSQI
Hugos et al. (2019) (America)	109/109	I:53.9 ± 9.8 C:53.6 ± 10.5	RRMS/SPMS/PPMS I:67/15/26 C:60/21/26	≤6.5	I:12.3 ± 7.6 C:12.7 ± 9.3	61/157	I: FTC C: Control	2-hour group sessions, lasted 6 weeks	PSQI
Kiropoulos et al. (2016) (Australia)	15/15	I:34.60 ± 9.06 C:39.27 ± 9.93	RRMS I:15 C:15	≤5.5	Months I: 26.20 ± 15.58 C: 23.53 ± 16.06	8/22	I: CBT C: Control	8 sessions (apart from the first session which was 1.5 h) were 1 h per week, lasted 8 weeks	PSQI
Li et al. (2022) (China)	I1:I2:C 39/37/20	I1: 20.56 ± 1.79 I2: 21.38 ± 2.13 C: 20.90 ± 1.41	NA	NA	NA	23/73	I1: Tele-CBT I2: Sleep hygiene C: Control	I1: once a day, each of about 10–15 min, lasted 3 weeks I2: sleep education, lasted 3 weeks	PSQI
Motl et al. (2023) (America)	124/124	I:48.8 ± 9.4 C:48.8 ± 9.5	NA	≤6.5	I:13.9 ± 8.7 C:11.4 ± 8.8	11/237	I: BT C: Control	behavioral intervention, lasted 6 months	PSQI
Grubić Kezele et al. (2023) (Croatia)	13/11	I:50.0 ± 9.3 C:53.8 ± 11.8	RRMS/SPMS/PPMS I:7/4/2 C:4/6/1	2.0–5.5	NA	10/14	I: Individualized exercise C: Control	2 times a week, 60 min/session, lasted 8 weeks	PSQI, ISI
Royer et al. (2024) (France )	12/13	I:52.0 ± 9.2 C:48.2 ± 9.2	NA	2.0–5.5	I:11.3 ± 5.0 C:10.2 ± 5.9	8/17	I: Individualized exercise C: AE+RT	I:60 min a sessions,3 times a week,lasted 12 weeks C: AE two times per week, RT at least two days per week, 36 training sessions	PSQI
Kavuran & Yurttaş (2024) (Turkey)	31/32	I:42.36 ± 13.25 C:44.27 ± 11.32	RRMS/SPMS/PPMS/PRMS I: 17/7/6/3 C:18/8/2/5	1.5–4.0	I:5.54 ± 1.73 C:7.56 ± 2.31	27/36	I: Aromatherapy C: Control	apply lavender oil for 30 days	PSQI
Cederberg & Motl (2021) (America)	7/7	I:55.60 ± 9.50 C:57.40 ± 13.00	RRMS/PPMS I:7/0 C:6/1	≤6.0	I:22.0 ± 4.4 C:20.0 ± 7.3	3/11	I: BT C: Control	12 one-on-one video chat sessions, lasted 8 weeks	PSQI
Açık et al. (2023) (Turkey)	I1:I2:C 16/11/16	I1:38.12 ± 9.11 I2:39.27 ± 10.45 C: 37.0 ± 9.47	NA	1.0–4.0	I1:6.5 ± 5.78 I2: 8.09 ± 6.33 C:7.69 ± 5.87	17/26	I1: AE I2: RT C: Control	I1: 30 min three days a week, lasted 3 months I2: strength exercise three days a week, including one set of 12–15 repetitions in the first month, two sets of 12–15 repetitions in the second month, and three sets of 12–15 repetitions in the third month, lasted 3 months	PSQI
Turkowitch et al. (2022) (America)	11/10	I:50.3 ± 13.5 C:51.1 ± 7.9	RRMS/SPMS I:11/0 C:8/2	≤6.0	NA	3/18	I1: Tele-CBT I2: CBT	once a week CBT sessions, lasted 6 weeks	PSQI, ISI
Sadeghi-Bahmani et al. (2022) (America)	I1:I2:C 26/25/25	I1:37.21 ± 9.83 I2:38.80 ± 6.01 C: 40.20 ± 10.72	NA	1.0–4.0	NA	14/62	I1: ACT I2: Mindfulness intervention C: Control	once a week for about 90–120 min, lasted 8 weeks	ISI
Siengsukon et al. (2020) (America )	I1:I2:C 10/10/10	I1:51.1 ± 7.9 I2:50.4 ± 12.4 C: 56.9 ± 10.1	RRMS/SPMS I1:8/2 I2:10/0 C:9/1	1.0–6.0	I1:17.3 ± 8.5 I2: 9.1 ± 8.9 C:18.3 ± 11.4	3/27	I1: CBT I2: ME C: Control	I1:once a week for 45–60 min, lasted 6 weeks I2: gentle stretching and self-selected light or sedentary activity once a week, lasted 6 weeks	PSQI, ISI
Sgoifo et al. (2017) (Italy)	24/24	NA	PPMS/RRMS/SPMS I:0/20/4 C:1/22/1	NA	I:8.2 ± 7.3 C:10.5 ± 8.5	NA	I: ME C: Control	ME intervention, 60 min/session for eight times a week, lasted 2 months	ISI
Sadeghi-Bahmani et al. (2019) (Switzerland)	I1:I2:I3 24/26/21	I1:39.17 ± 8.66 I2:37.96 ± 8.69 C: 37.90 ± 9.91	NA	1.0–4.0	I1:8.13 ± 6.3 I2:6.92 ± 6.81 C: 7.21 ± 6.57	NA	I1: ME I2: RT C: Control	I1: 30–45 min/each ME , three times a week, lasted 8weeks I2: 30–45 min/session RT, three times a week, lasted 8 weeks	ISI
Sun, Sun & Wang (2014) (China)	28/28	I:40.71 ± 5. 75 C:40.21 ± 5.92	NA	NA	I:10.39 ± 2.51 C:10.54 ± 2.77	27/29	I: Music therapy C: Control	30 min each, once a day, lasted 4 weeks	PSQI
Khadadah et al. (2022) (Canada)	17/17	I:49.60 ± 10.00 C:52.80 ± 8.80	RRMS/SPMS I:14/3 C:13/4	≤6.5	I:21.2 ± 10.1 C:19.8 ± 11	12/22	I: CPAP C: Control	use CPAP nightly for 6 months	PSQI
Mazerolle et al. (2024) (Canada )	16/12	I:49.9 ± 9.4 C:53.0 ± 9.5	RRMS/SPMS/ IMT I:9/5/2 C:9/1/2		I:22.3 ± 9.5 C:22.4 ± 13.2	9/19	I: CPAP C: Control	use CPAP 5.4 ± 1.0 h/night for 6 months	PSQI
Karakas et al. (2024) (Turkey)	16/16	Median (IQR) I: 37.0 (32.5, 40.0) C: 37.5 (30.2,43.7)	NA	2.0–5.0	I: 10.3 ± 7.1 C: 7.75 ± 4.8	8/24	I: ME C: Control	8-week GMI training period, the first 2-weeks involved implicit MI training while 6-weeks explicit MI training were conducted	PSQI
Sehat-Nejad et al. (2021) (Iran)	I1:I2:C 30/30/30	I1: 33.17 ± 7.51 I2: 32.7 ± 8.15 C: 20.90 ± 1.41	NA	NA	I1: 6.6 ± 5.32 I2: 6.27 ± 3.85 C: 6.1 ± 3.79	49/41	I1: Reflexology I2: Effleurage C: Control	I1: reflexology lasted for 40 min (20 min for each foot) twice a week ,lasted eight weeks I2: effleurage massage lasted for 40 min twice a week, lasted eight weeks	ISI
Asghari et al. (2025) (Iran)	I1:I2:C 10/8/10	I1:40.00 ± 6.63 I2:36.25 ± 9.98 C:34.10 ± 4.72	RRMS (all)	NA	I1:11.50 ± 3.95 I2:10.38 ± 2.62 C:10.10 ± 2.28	8/20	I1: Individualized exercise I2: ME C: Control	3 sessions/week, 30–60 min/session, 8 weeks	PSQI
Kaya et al. (2025) (Türkiye)	I1:I2 15/14	I1:37.5 ± 13.0 I2:41.7 ± 10.2	NA	≤4.0	NA	9/20	I1: Individualized exercise I2: RT	I1: online supervised exercise for 40 min, 2 days/week for 12 weeks I2:Control group: home-based video exercise with the same program, 2 days/week for 12 weeks with weekly phone calls	PSQI
Mansourzadeh et al. (2023) (Iran)	53/53	I: 36.55 ± 7.36 C: 35.91 ± 7.76	NA	1.0–4.5	NA	21/85	I: CBT C: Control	20 weekly group schema therapy sessions, each lasting 90 min	PSQI
Khodaie et al. (2025) (Iran)	31/31	I: 38.10 ± 7.35 C: 40.16 ± 9.26	RRMS(all)	1.0–3.5	I: 8.87 ± 4.11 C: 9.65 ± 6.35	12/50	I: Reflexology C: Control	Twice a week for 12 weeks; needle retention: 30 min; points: GV 20, EX-HN 1, GV 24, GV 29, GB 20, CV 12, ST 25, CV 6, HT 7, PC 6, ST 36, SP 6, LR 3	PSQI
Masoudi et al. (2022) (Iran)	35/32	I: 38.23 ± 7.73 C: 39.16 ± 7.46	NA	NA	I: 22 (≤5 years) 5 (6–9 years) 8 (≥10 years) C: 20 (5 years) 5 (6–9 years) 7 (≥10 years)	12/58	I: CBT C: Control	8 sessions, 60 min each, twice a week; 14 principles of Fordyce Happiness Program; group discussions in 7-member groups	PSQI

**Notes.**

I intervention C control M male F female IQR interquartile range RRMS relapsing remitting multiple sclerosis SPMS secondary progressive multiple sclerosis PPMS primary progressive multiple sclerosis PRMS progressive multiple sclerosis IMT immunomodulating treatment NA not applicable AE aerobic exercise RT resistance training ME mind-body exercise BLT bright light therapy OT occupational therapy CBT cognitive behavioural therapy BT behavioural therapy FTC fatigue: take control ACT acceptance and commitment therapy CPAP continuous positive airway pressure PSQI Pittsburgh Sleep Quality Index ISI Insomnia Severity Index

### Results of traditional meta-analysis

**Figure 3:**
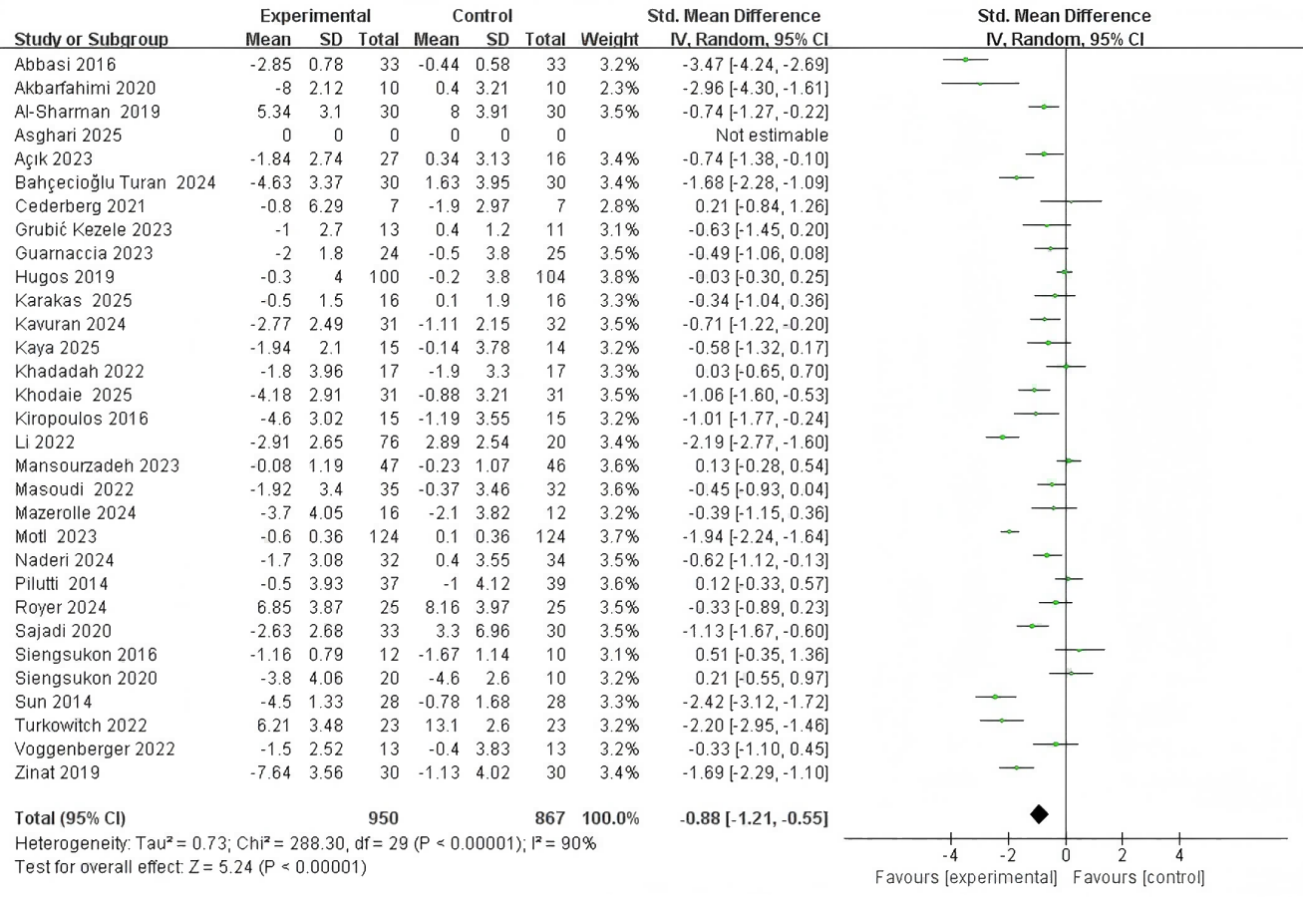
Effect of non-pharmacological interventions on PSQI in MS.

**Figure 4:**
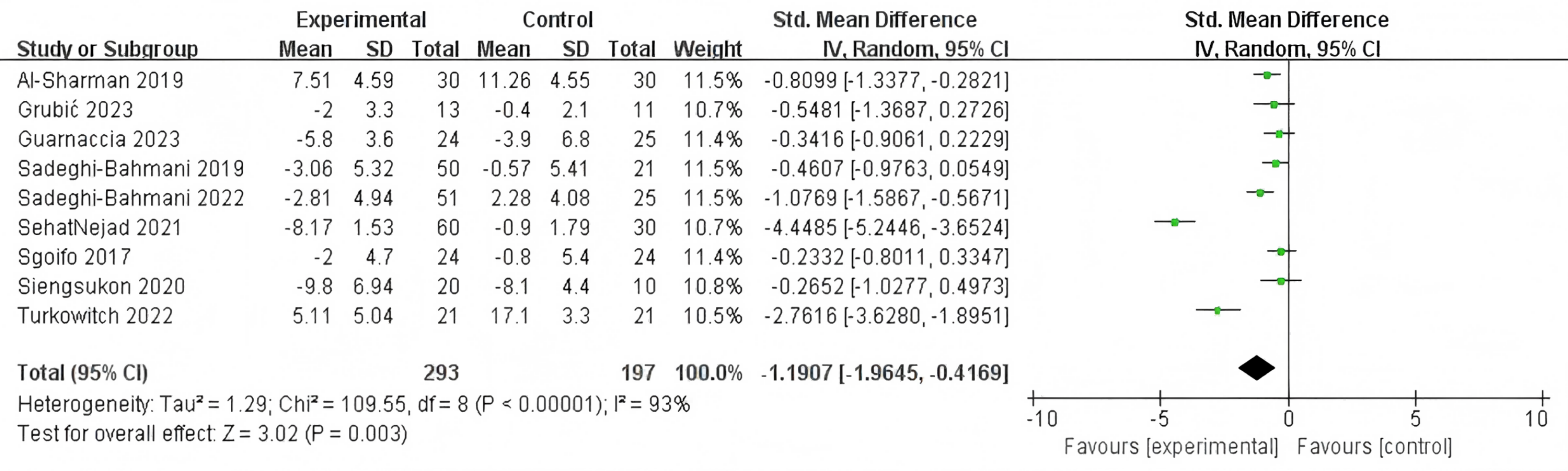
Effect of non-pharmacological interventions on ISI in MS.

Forest plots illustrate a contribution analysis of non-pharmacological intervention measures to improve sleep quality in patients with MS (Figs. 3–4). The combination meta-analysis in PSQI revealed high heterogeneity (I^2^ = 90%, *P* < 0.001). Therefore, a random effect model was applied. The combination effect estimate was (SMD = −0.88, 95% CI [−1.21 to −0.55], *P* <  0.001), and the combined meta-analysis in ISI revealed high heterogeneity (I^2^ = 93%, *P* < 0.001). Therefore, a random effect model was utilized to test the effect. The combined effect size was (SMD = −1.19, 95% CI [−1.96 to −0.42], *P* < 0.001), indicating that the effect was statistically significant; non-pharmacological intervention measures can significantly improve the sleep quality of patients with MS.

### Results of NMA

**Figure 5:**
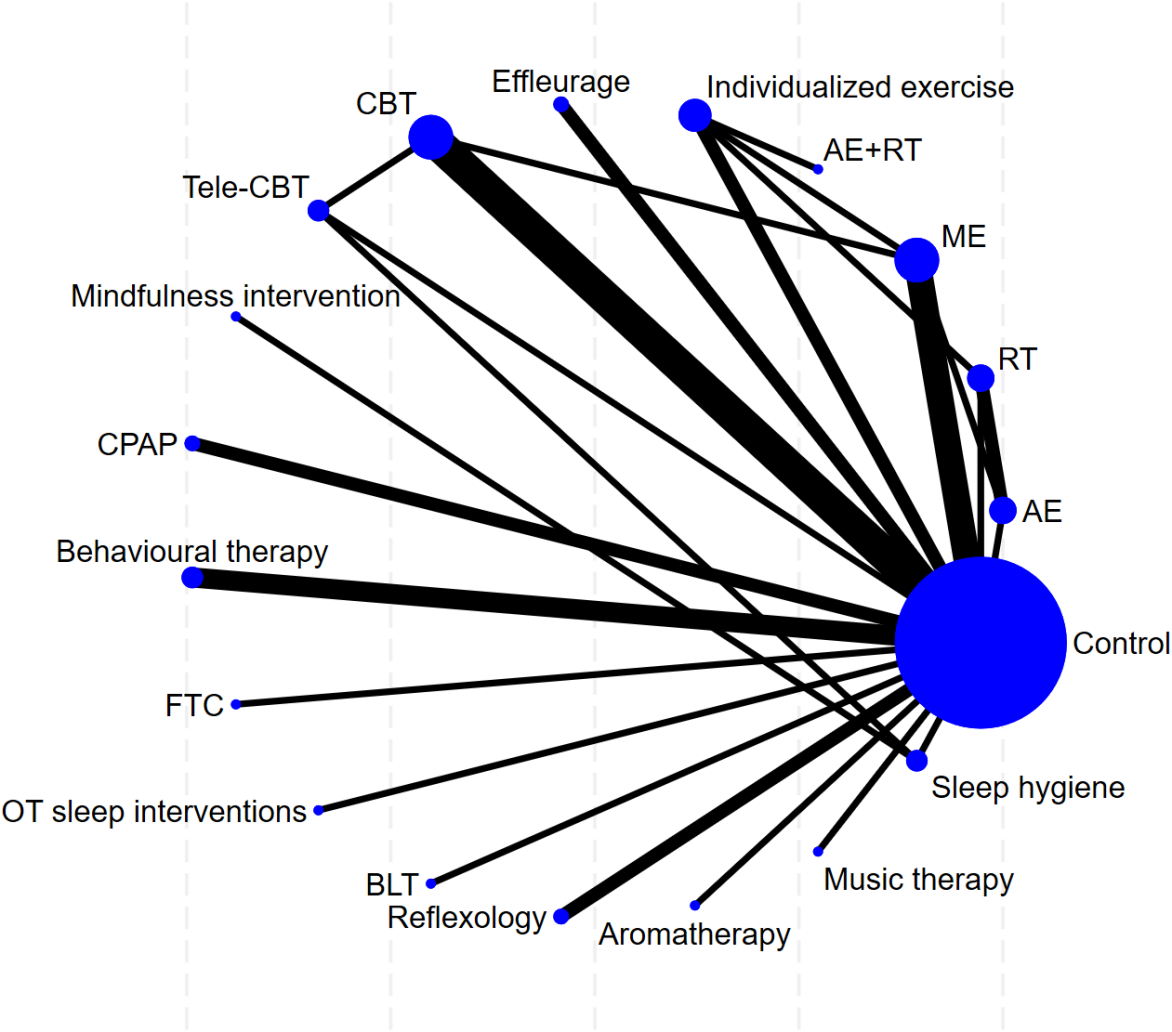
The network map of non-pharmacological interventions for PSQI in MS.

**Figure 6:**
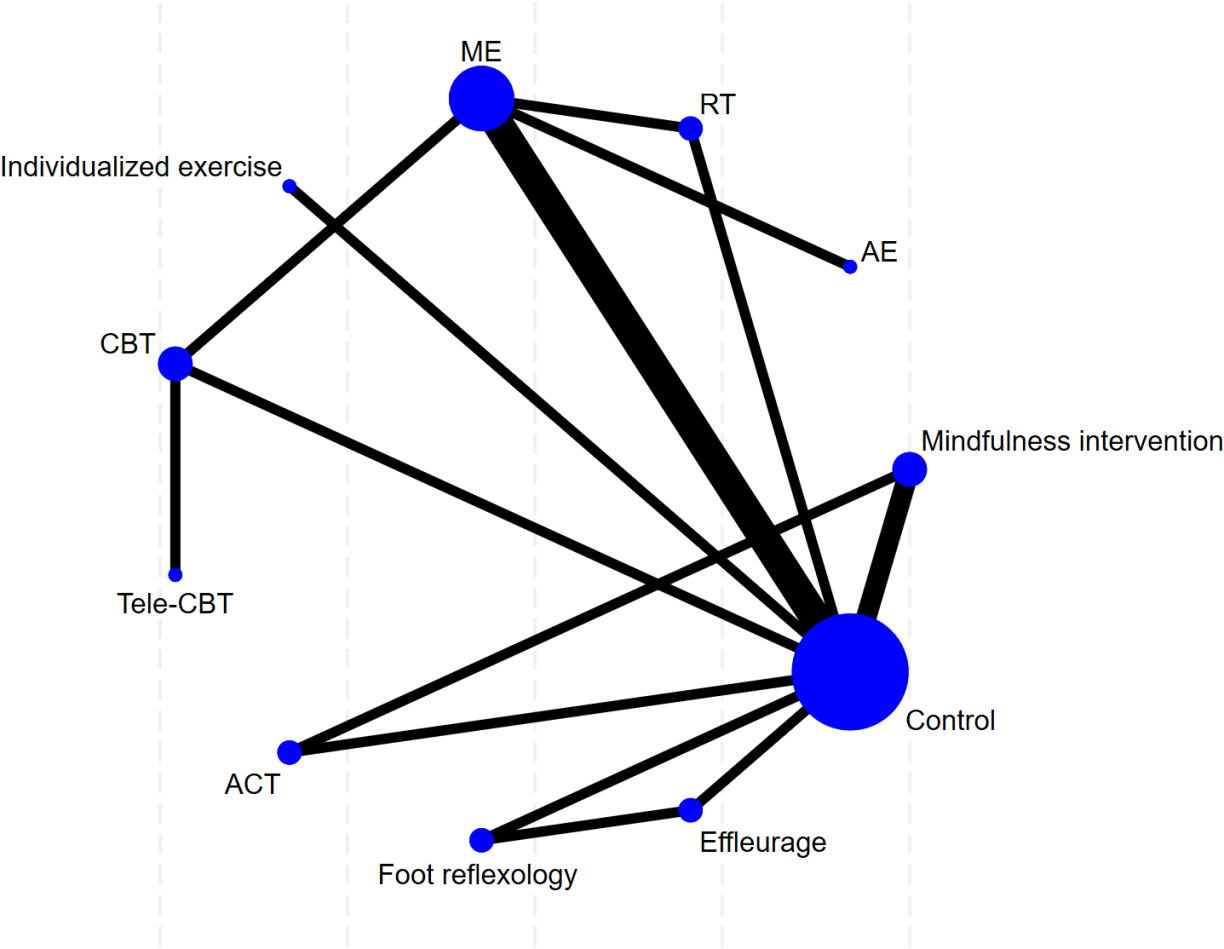
The network map of non-pharmacological interventions for ISI in MS.

This study included 35 randomized controlled trials, comprising two primary outcome measures: PSQI and ISI. Separate network meta-analyses were conducted for each outcome measure across the included trials. For each indicator, a dedicated network plot was constructed. These plots intuitively illustrate direct and indirect comparisons between different interventions, and indicate the number of studies contributing to each comparison. These specific results are depicted in Figs. 5–6.

The two datasets were evaluated for overall and local inconsistency, respectively, with *P* > 0.05, indicating that the overall and local inconsistencies were non-significant and the consistency was good. Consequently, this study adopted the consistency model analysis.

### Main outcome: PSQI

In total, 31 studies included PSQI as an outcome indicator. The NMA results revealed that individualized exercise [−3.05, 95% CI [−5.79 to −0.31]], effleurage [−4.24, 95% CI [−7.36 to −1.12]], CBT [−2.47, 95% CI [−4.30 to −0.63]], Tele-CBT [−4.37, 95% CI [−7.67 to −1.07]], mindfulness intervention [−6.96, 95% CI [−12.93 to −1.00]], OT-based sleep interventions [−8.40, 95% CI [−13.10 to −3.70]], foot reflexology [−4.47, 95% CI [−7.70 to −1.24]] and sleep hygiene [−5.46, 95% CI [−9.52 to −1.40]] exhibited better improvement effects on the sleep quality of patients with MS (Table 2). The top five probabilities of SUCRA are as follows: OT sleep interventions (SUCRA = 94.2%), mindfulness intervention (SUCRA = 85.9%), sleep hygiene (SUCRA = 78.6%), foot reflexology (SUCRA = 71.5%), and Tele-CBT (SUCRA = 70.0%) (Table 3).

**Table 2:** League table for non-pharmacologic interventions in PSQI.

AE					
0.20 (−2.63, 3.03)	RT	□	□	□	
−1.24 (−4.44, 1.97)	−1.44 (−4.82, 1.95)	ME	□	□	
1.66 (−4.55, 7.88)	1.47 (−4.57, 7.51)	2.90 (−3.01, 8.82)	AE+RT	□	
1.27 (−2.36,4.89)	1.07 (−2.26, 4.39)	2.50 (−0.58, 5.59)	−0.40 (−5.44, 4.64)	Individualized exercise	
2.46 (−1.95, 6.86)	2.26 (−2.16, 6.68)	3.70 (−0.03, 7.42)	0.79 (−5.74, 7.33)	1.19 (−2.96, 5.35)	Effleurage
0.68 (−2.87, 4.24)	0.49 (−3.11, 4.08)	1.92 (−0.67, 4.51)	−0.98 (−6.99, 5.03)	−0.58 (−3.85, 2.68)	−1.77 (−5.39, 1.85)
2.59 (−1.93, 7.10)	2.39 (−2.15, 6.93)	3.83 (−0.00, 7.66)	0.92 (−5.69, 7.54)	1.32 (−2.96, 5.61)	0.13 (−4.41, 4.68)
5.18 (−1.54, 11.90)	4.98 (−1.75, 11.71)	6.42 (0.13, 12.70)	3.51 (−4.76, 11.79)	3.91 (−2.65, 10.47)	2.72 (−4.01, 9.45)
−1.08 (−5.71, 3.55)	−1.28 (−5.93, 3.37)	0.16 (−3.83, 4.15)	−2.75 (−9.44, 3.94)	−2.35 (−6.74, 2.05)	−3.54 (−8.18, 1.11)
−1.85 (−5.98, 2.28)	−2.04 (−6.19, 2.11)	−0.61 (−4.00, 2.79)	−3.51 (−9.87, 2.84)	−3.11 (−6.98, 0.75)	**−4.30 (−8.45, −0.16)**
−1.68 (−6.90, 3.53)	−1.88 (−7.11, 3.35)	−0.45 (−5.10, 4.21)	−3.35 (−10.46, 3.76)	−2.95 (−7.96, 2.06)	−4.14 (−9.37, 1.08)
6.62 (0.98, 12.25)	6.42 (0.77, 12.06)	7.85 (2.74, 12.97)	4.95 (−2.47, 12.37)	5.35 (−0.09, 10.79)	4.16 (−1.48, 9.80)
−0.68 (−6.36, 4.99)	−0.88 (−6.57, 4.81)	0.55 (−4.61, 5.72)	−2.35 (−9.80, 5.10)	−1.95 (−7.44, 3.54)	−3.14 (−8.83, 2.55)
2.68 (−1.79, 7.16)	2.49 (−2.01, 6.98)	3.92 (0.11, 7.74)	1.02 (−5.57, 7.60)	1.42 (−2.82, 5.65)	0.23 (−4.26, 4.72)
−0.12 (−5.35, 5.10)	−0.32 (−5.57, 4.92)	1.11 (−3.56, 5.79)	−1.79 (−8.91, 5.33)	−1.39 (−6.41, 3.63)	−2.58 (−7.82, 2.66)
1.94 (−3.23, 7.10)	1.74 (−3.44, 6.92)	3.17 (−1.42, 7.77)	0.27 (−6.80, 7.34)	0.67 (−4.28, 5.62)	−0.52 (−5.70, 4.65)
3.68 (−1.42, 8.78)	3.48 (−1.64, 8.60)	4.92 (0.40, 9.43)	2.01 (−5.01, 9.04)	2.41 (−2.48, 7.30)	1.22 (−3.90, 6.34)
−1.78 (−4.89, 1.32)	−1.98 (−5.11, 1.15)	−0.55 (−2.57, 1.48)	−3.45 (−9.19, 2.29)	**−3.05 (−5.79, −0.31)**	**−4.24 (−7.36, −1.12)**
CBT	□	□	□		
1.91 (−1.46, 5.27)	Tele-CBT	□	□		
4.49 (−1.63, 10.61)	2.59 (−3.35, 8.52)	Mindfulness intervention	□		
−1.76 (−5.66, 2.13)	−3.67 (−8.44, 1.10)	−6.26 (−13.14, 0.63)	CPAP		
−2.53 (−5.82, 0.76)	**−4.43 (−8.71, −0.16)**	**−7.02 (−13.58, −0.47)**	−0.77 (−5.15, 3.62)	Behavioural therapy	
−2.37 (−6.94, 2.20)	−4.27 (−9.61, 1.06)	−6.86 (−14.15, 0.43)	−0.60 (−6.02, 4.81)	0.16 (−4.83, 5.16)	FTC
5.93 (0.89, 10.98)	4.03 (−1.72, 9.77)	1.44 (−6.16, 9.03)	7.70 (1.87, 13.52)	8.46 (3.03, 13.89)	8.30 (2.01, 14.59)
−1.37 (−6.46, 3.73)	−3.27 (−9.06, 2.52)	−5.86 (−13.49, 1.77)	0.40 (−5.47, 6.26)	1.16 (−4.32, 6.64)	1.00 (−5.34, 7.34)
2.00 (−1.71, 5.71)	0.09 (−4.52, 4.71)	−2.49 (−9.28, 4.29)	3.76 (−0.95, 8.48)	4.53 (0.30, 8.75)	4.37 (−0.92, 9.66)
−0.81 (−5.40, 3.78)	−2.71 (−8.06, 2.64)	−5.30 (−12.60, 2.00)	0.96 (−4.48, 6.39)	1.72 (−3.29, 6.73)	1.56 (−4.38, 7.50)
1.25 (−3.26, 5.77)	−0.65 (−5.94, 4.63)	−3.24 (−10.50, 4.01)	3.02 (−2.35, 8.39)	3.78 (−1.16, 8.73)	3.62 (−2.26, 9.50)
2.99 (−1.29, 7.27)	1.09 (−2.92, 5.10)	−1.50 (−5.87, 2.87)	4.76 (−0.56, 10.08)	5.52 (0.64, 10.41)	5.36 (−0.47, 11.19)
**−2.47 (−4.30, −0.63)**	**−4.37 (−7.67, −1.07)**	**−6.96 (−12.93, −1.00)**	−0.70 (−4.14, 2.73)	0.06 (−2.66, 2.78)	−0.10 (−4.29, 4.09)

OT sleep interventions	□	□	□			
**−7.30 (−13.98, −0.62)**	BLT	□	□			
−3.93 (−9.63, 1.77)	3.37 (−2.38, 9.11)	Reflexology	□			
**−6.74 (−13.05, −0.43)**	0.56 (−5.79, 6.91)	−2.81 (−8.11, 2.50)	Aromatherapy			
−4.68 (−10.93, 1.57)	2.62 (−3.68, 8.91)	−0.75 (−5.99, 4.49)	2.06 (−3.83, 7.95)	Music therapy	□	□
−2.94 (−9.15, 3.27)	4.36 (−1.89, 10.61)	0.99 (−4.19, 6.18)	3.80 (−2.04, 9.65)	1.74 (−4.04, 7.53)	Sleep hygiene	□
**−8.40 (−13.10, −3.70)**	−1.10 (−5.85, 3.65)	**−4.47 (−7.70, −1.24)**	−1.66 (−5.87, 2.55)	−3.72 (−7.85, 0.41)	**−5.46 (−9.52, −1.40)**	Contol

**Notes.**

Bold indicates significant differences in results.

**Table 3:** SUCRA values for non-pharmacologic interventions in PSQI.

Treatments	SUCRA
AE	39.1
RT	42.1
ME	22.4
AE+RT	58.1
Individualized exercise	56.4
Effleurage	69.5
CBT	48.6
Tele-CBT	70.0
Mindfulness intervention	85.9
CPAP	26.5
Behavioural therapy	17.0
FTC	21.7
OT sleep interventions	94.2
BLT	33.2
Reflexology	71.5
Aromatherapy	38.3
Music therapy	62.3
Sleep hygiene	78.6
Control	14.6

### Main outcome: ISI

In total, nine studies included ISI as an outcome indicator. The results of the NMA demonstrated that compared with the conventional treatment, mindfulness intervention [−2.94, 95% CI [−4.71 to −1.16]], aerobic exercise [−5.72, 95% CI [−9.58 to −1.87]], CBT [−6.98, 95% CI [−11.23 to −2.72]], acceptance and commitment therapy [−6.34, 95% CI [−8.89 to −3.80]], foot reflexology [−6.90, 95% CI [−7.76 to −6.04]], and effleurage [−7.63, 95% CI [−8.43 to −6.83]] exhibited better improvement effects on the sleep quality of patients with MS (Table 4). Table 5 presents the top five probabilities of SUCRA: effleurage (SUCRA = 91.9%), CBT (SUCRA = 80.1%), foot reflexology (SUCRA = 77.0%), acceptance and commitment therapy (SUCRA = 72.1%), and aerobic exercise (SUCRA = 66.4%).

**Table 4:** League table for non-pharmacologic interventions in ISI.

Mindfulness intervention	□	□	□	□	□					
2.79 (−1.46, 7.03)	AE	□	□	□	□					
−1.57 (−4.87, 1.73)	**−4.36 (−8.67, −0.04)**	RT	□	□	□					
−1.81 (−4.43, 0.80)	**−4.60 (−7.94, −1.26)**	−0.24 (−2.98,2.49)	ME	□	□					
−1.34 (−4.15, 1.48)	−4.12 (−8.55, 0.30)	0.23 (−3.30, 3.76)	0.48 (−2.43, 3.38)	Individualized exercise	□					
4.04 (−0.57, 8.65)	1.25 (−4.24, 6.75)	5.61 (0.67, 10.55)	5.85 (1.49, 10.22)	5.38 (0.60, 10.16)	CBT					
1.54 (−4.29, 7.37)	−1.25 (−7.80, 5.31)	3.11 (−2.98, 9.20)	3.35 (−2.28, 8.99)	2.88 (−3.09, 8.84)	−2.50 (−6.07, 1.07)	Tele-CBT	□	□	□	□
3.40 (0.90, 5.91)	0.62 (−4.00, 5.23)	4.97 (1.20, 8.74)	5.22 (2.03, 8.40)	4.74 (1.39, 8.09)	−0.64 (−5.59, 4.32)	1.86 (−4.25, 7.97)	ACT	□	□	□
3.96 (1.99, 5.94)	1.18 (−2.77, 5.12)	5.53 (2.62, 8.44)	5.78 (3.67, 7.88)	5.30 (2.95, 7.65)	−0.08 (−4.42, 4.26)	2.42 (−3.20, 8.04)	0.56 (−2.13, 3.25)	Reflexology	□	□
4.69 (2.74, 6.64)	1.91 (−2.03, 5.84)	6.26 (3.37, 9.16)	6.51 (4.43, 8.59)	6.03 (3.71, 8.35)	0.65 (−3.68, 4.98)	3.15 (−2.46, 8.76)	1.29 (−1.38, 3.96)	0.73 (−0.03, 1.49)	Effleurage	□
**−2.94 (−4.71, −1.16)**	**−5.72 (−9.58, −1.87)**	−1.37 (−4.15, 1.41)	−1.12 (−3.04, 0.79)	−1.60 (−3.78, 0.58)	**−6.98 (−11.23, −2.72)**	−4.48 (−10.03, 1.07)	**−6.34 (−8.89, −3.80)**	**−6.90 (−7.76, −6.04)**	**−7.63 (−8.43, −6.83)**	Control

**Notes.**

Bold indicates significant differences in results.

**Table 5:** League table for non-pharmacologic interventions in ISI.

Treatments	SUCRA
Mindfulness intervention	40.2
AE	66.4
RT	22.3
ME	19.0
Individualized exercise	25.0
CBT	80.1
Tele-CBT	52.5
ACT	72.1
Reflexology	77.0
Effleurage	91.9
Control	4.3

### Analysis of publication bias

**Figure 7:**
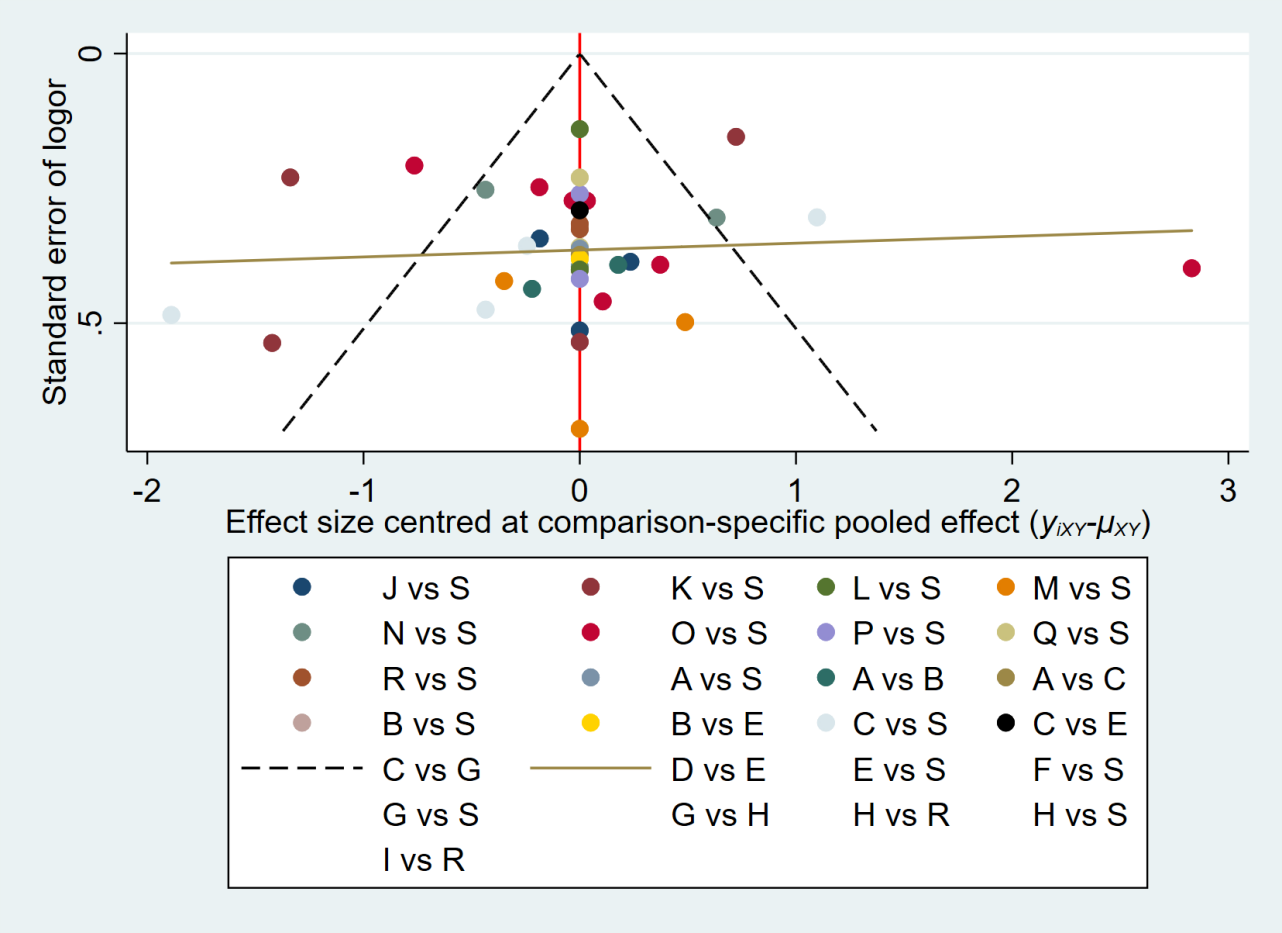
The funnel plot of non-pharmacological interventions for PSQI in MS.

Figure 7 depicts the publication bias analysis for the same outcome measures across ≥ 10 articles based on the PSQI score. The results exhibited a generally symmetrical distribution, indicating a relatively minor publication bias. Egger’s test revealed no publication bias (*P* = 0.094).

## Discussion

This NMA evaluated the comparative efficacy of 20 non-pharmacological interventions for improving sleep outcomes in patients with MS. The SUCRA ranking indicated that OT-based sleep interventions (SUCRA = 94.2%) ranked the highest for improving overall sleep quality, whereas effleurage (SUCRA = 91.9%) ranked highest for alleviating insomnia symptoms. Other interventions, including sleep hygiene, cognitive behavioral therapy (remote and face-to-face), and mindfulness, demonstrated positive effects.

For PSQI, which reflects the overall sleep quality, 31 articles analyzed this indicator. The findings confirmed that numerous non-pharmacological interventions contribute to varying degrees in improving sleep quality among patients with MS. Among them, OT-based sleep interventions (SUCRA = 94.2%) emerged as the most effective intervention. The superior performance of OT-based sleep interventions in our analysis is corroborated by a recent systematic review, which confirmed that OT-based sleep interventions effectively address sleep and activities of daily living in patients with MS (Yu, Foidel & Tomlin, 2025). The principal strength of OT-based sleep interventions lies in their comprehensive methodology. Rather than a singular intervention, it constitutes a multidimensional and synergistic approach that systematically combines non-pharmacological strategies, including exercise, sleep hygiene, and cognitive behavioral therapy, within the evidence-based Person-Environment-Occupation practice framework, hereby addressing the multifactorial nature of sleep disturbances in MS, which often intertwine with fatigue, pain, and daytime dysfunction (Leland et al., 2014; Hugos et al., 2019). Previous studies confirmed the beneficial effects of exercise, sleep hygiene, and cognitive behavioral therapy on improving sleep quality in patients with MS (Abbasi, Alimohammadi & Pahlavanzadeh, 2016; Siengsukon et al., 2016; Guarnaccia et al., 2023).

The research effect has been validated in Parkinson’s disease (PD) (Daftary & Dedhiya, 2024). The proposed mechanisms include re-establishment of sleep rhythms through behavioral and environmental regulation (Doucet, Franc & Hunter, 2021), optimization of daytime functioning to generate positive feedback for nighttime sleep (Fan & Drumheller, 2021), and managing PD-specific motor symptoms. Compared with research on MS, OT-based sleep interventions exhibited transdiagnostic efficacy in improving fatigue and quality of life in patients with neurological disorders, sharing core mechanisms of behavioral regulation and activity balancing. The main difference lies in disease-specific symptom management: MS interventions focus more on pain and temperature regulation, whereas PD interventions emphasize motor symptom management and circadian rhythm restoration. Future research should further investigate the common mechanisms and personalized strategies of OT-based sleep interventions across different neurological diseases and may further decompose the contributions of each component within OT-based sleep intervention protocols to optimize their precise application in sleep disorders among patients with MS.

Regarding ISI, which measures the severity and impact of insomnia, effleurage was the most effective (SUCRA = 91.9%). This study aligns with a previous study in the PD population (Tang et al., 2024), indicating that effleurage interventions exhibit distinct advantages in improving sleep across diverse neurological populations. This indicates a potential cross-disease common mechanism of action. Effleurage, as a complementary therapy, exhibited significant advantages in various fields utilizing mild to moderate stress techniques for patient populations in different medical environments (Gaballah, El-Deen & Hebeshy, 2023). The medium pressure applied during the massage may enhance the vagus nerve activity and inhibit the lower midbrain-pituitary-adrenal function by stimulating the edge system of the final signal, which has resulted in decreased adrenaline and cortisol levels and high serum levels (Field, 2016). The level of bolstered adrenaline reduces the slow heart rate and blood pressure and improves sleep (Blagrove et al., 2012). The increased level of serotonin can ameliorate depression symptoms and relieve pain, thereby improving overall sleep quality. Additionally, massage usually produces better results than exercise, especially in patients with MS, who may be exhausted (Field, 2016). However, many studies have demonstrated that the effect of massage therapy on the sleep quality of patients with MS is limited.

Furthermore, foot reflexology, a type of massage that utilizes thumb and finger techniques to stimulate specific foot areas, has demonstrated notable efficacy. Many scholars believe that vitality, or life energy, flows through the foot passages to all body organs, and any blockage will lead to disease. Stimulating reflex points on the feet can alleviate blockages in these flow paths; reflex therapy eliminates these blockages, enhances energy flow in these channels, and improves the health condition of patients. Reflex therapy can improve the sleep quality of patients with insomnia by alleviating stress and tension and maintaining body balance, or homeostasis (Sehat-Nejad et al., 2021). This NMA demonstrates that acceptance and commitment therapy and mindfulness interventions exert favorable effects. Although both share the components of “acceptance” and “awareness,” their mechanisms of action are different. Acceptance and commitment therapy focuses more on reconstructing the correlation between patients’ sleep and disease symptoms through cognition and value-oriented behaviors, rather than directly targeting sleep physiology (Hayes, 2019); however, mindfulness improves sleep quality indirectly by enhancing non-judgmental awareness of present-moment experiences, reducing rumination and pre-sleep anxiety (Simpson et al., 2019).

Herein, psychological interventions ranked relatively lower than effleurage interventions. This may reflect that in MS, which is characterized by prominent physical symptoms and fatigue, direct physical interventions that combine physiological relaxation and emotional support are more likely to be accepted by patients and take effect more quickly. Compared with previous studies, this research found that exercise interventions (aerobic exercise and individualized exercise) have a definite effect on improving sleep in patients with MS. However, the efficacy of exercise interventions may be moderated by the degree of disability, fatigue level, and individual preferences, which may explain why they did not rank among the top. Future research should implement targeted interventions based on different MS subtypes, disability levels, and sleep disorder types so as to facilitate personalized interventions for different groups of patients with MS.

### Limitations and future research

This study has several limitations. First, this study relied on patient-reported outcomes such as the PSQI and ISI, which are validated and sensitive measures of perceived sleep quality. However, as patient-reported outcomes cannot assess physiological sleep architecture, our findings focused primarily on improvements in subjective sleep experience. Future trials should incorporate objective measures such as actigraphy and polysomnography to complement these data. Second, our inclusion criteria did not require participants to have baseline sleep disturbances. Although this allowed us to assess the broader potential of interventions for improving and preventing sleep problems in the MS population, it may have diluted the observed effect sizes for patients with clinically significant sleep disorders. Future research could focus specifically on patients with MS with diagnosed sleep conditions to assess targeted treatment efficacy. Third, variability in MS subtypes, disease severity, and intervention protocols might have influenced outcomes. Fourth, we comprehensively examined non-pharmacological interventions for sleep disorders in patients with MS; however, the language was limited to Chinese and English, which might lead to selection bias. Furthermore, the overall certainty of evidence was low to moderate for most comparisons. Future high-quality randomized controlled trials should focus on understudied modalities, standardized outcome measures, and longer follow-up periods to strengthen clinical recommendations.

## Conclusion

This NMA demonstrated that non-pharmacological interventions, especially occupational therapy-based sleep interventions and effleurage massage, can improve sleep quality and ameliorate insomnia symptoms in people with MS. These approaches should be considered as part of comprehensive sleep management. Further research is needed to validate these findings, especially for interventions with limited current evidence, and to investigate the integration of objective sleep assessments and patient-centered outcome measures.

##  Supplemental Information

10.7717/peerj.20900/supp-1 Supplemental Information 1 PRISMA checklist

10.7717/peerj.20900/supp-2 Supplemental Information 2 Search strategy in each database

10.7717/peerj.20900/supp-3 Supplemental Information 3 Test of consistency

10.7717/peerj.20900/supp-4 Supplemental Information 4 The SUCRA plot for non-pharmacologic interventions in PSQI

10.7717/peerj.20900/supp-5 Supplemental Information 5 The SUCRA plot for non-pharmacologic interventions in ISI

## Additional Information and Declarations

### Data Availability

The following information was supplied regarding data availability: This is a systematic review/meta-analysis.
