# Supplementary material for: Comparative efficacy of non- pharmacological interventions on sleep quality in patients with multiple sclerosis: a systematic review and network meta-analysis
Source: PeerJ. 2026 Mar 16;14:e20900. doi: 10.7717/peerj.20900 (PMC13001664; doi:10.7717/peerj.20900)
Supplement: Supplemental Information 2 [file peerj-14-20900-s002.docx]

| **Database** | **Search Strategy** |
| --- | --- |
| PubMed | (((("Multiple Sclerosis"[Mesh]) OR ((((((Sclerosis, Multiple[Title/Abstract]) ) OR (MS (Multiple Sclerosis[Title/Abstract]))) OR (Sclerosis, Disseminated[Title/Abstract])) OR (Disseminated Sclerosis[Title/Abstract])) OR (Multiple Sclerosis, Acute Fulminating[Title/Abstract]))) AND ((((((((((((((((((((((((((((((non-pharmacological[Title/Abstract]) OR (non-pharmaceutical[Title/Abstract])) OR (non-drug intervention[Title/Abstract])) OR (treatment[Title/Abstract])) OR (training[Title/Abstract])) OR (rehabilitation[Title/Abstract])) OR (exercise[Title/Abstract])) OR (diet[Title/Abstract])) OR (therapy[Title/Abstract])) OR (bright light therapy[Title/Abstract])) OR (physical therapy[Title/Abstract])) OR (group therapy[Title/Abstract])) OR (repetitive transcranial magnetic stimulation[Title/Abstract])) OR (deep brain stimulation[Title/Abstract])) OR (cognitive behavioral therapy[Title/Abstract])) OR (mindfulness meditation[Title/Abstract])) OR (Baduanjin[Title/Abstract])) OR (Qigong[Title/Abstract])) OR (continuous positive airway pressure[Title/Abstract])) OR (Tai Chi[Title/Abstract])) OR (acupuncture[Title/Abstract])) OR (massage therapy[Title/Abstract])) OR (muscle relaxation[Title/Abstract])) OR (yoga[Title/Abstract])) OR (dance[Title/Abstract])) OR (musicotherapy[Title/Abstract])) OR (behavioral cognitive therapy[Title/Abstract])) OR (psychotherapy[Title/Abstract])) OR (assistive technique[Title/Abstract])) OR (virtual reality[Title/Abstract]))) AND ((((((((dyssomnias[Title/Abstract]) OR (insomnia[Title/Abstract])) OR (agrypnia[Title/Abstract])) OR (sleep*[Title/Abstract])) OR (sleep disorders[Title/Abstract])) OR (sleepiness[Title/Abstract])) OR (sleep quality[Title/Abstract])) OR (sleep parasomnias[Title/Abstract]))) AND (("Randomized Controlled Trial" [Publication Type] OR "Randomized Controlled Trials as Topic"[Mesh] OR "Controlled Clinical Trial" [Publication Type]) OR ((Randomized[Title/Abstract]) OR (Placebo[Title/Abstract]))) |
| Embase | ('multiple sclerosis'/exp OR 'multiple sclerosis, acute fulminating':ab,ti OR 'multiple sclerosis':ab,ti OR 'sclerosis, disseminated':ab,ti OR 'disseminated sclerosis':ab,ti）AND （'non pharmacological':ab,ti OR 'non pharmaceutical':ab,ti OR 'non-drug intervention':ab,ti OR treatment:ab,ti OR training:ab,ti OR rehabilitation:ab,ti OR exercise:ab,ti OR diet:ab,ti OR therapy:ab,ti OR 'bright light therapy':ab,ti OR 'physical therapy':ab,ti OR 'group therapy':ab,ti OR 'repetitive transcranial magnetic stimulation':ab,ti OR 'deep brain stimulation':ab,ti OR 'cognitive behavioral therapy':ab,ti OR 'mindfulness meditation':ab,ti OR baduanjin:ab,ti OR qigong:ab,ti OR 'continuous positive airway pressure':ab,ti OR 'tai chi':ab,ti OR acupuncture:ab,ti OR 'massage therapy':ab,ti OR 'muscle relaxation':ab,ti OR yoga:ab,ti OR dance:ab,ti OR musicotherapy:ab,ti OR 'behavioral cognitive therapy':ab,ti OR psychotherapy:ab,ti OR 'assistive technique':ab,ti OR 'virtual reality':ab,ti）AND （'dyssomnia'/exp OR 'dyssomnia' OR 'sleep disorder'/exp OR 'sleep disorder' OR insomnia:ab,ti OR agrypnia:ab,ti OR sleepiness:ab,ti OR 'sleep quality':ab,ti OR 'sleep parasomnias':ab,ti） AND ('randomized controlled trial'/exp OR 'randomized controlled trial' OR 'controlled clinical trial (topic)'/exp OR 'controlled clinical trial (topic)' OR RCT OR 'placebo'/exp OR 'placebo' OR 'placebo':ab,ti OR randomized:ab,ti） |
| Web of Science | (Multiple Sclerosis OR Sclerosis, Multiple OR Sclerosis, Disseminated OR Disseminated Sclerosis OR MS (Multiple Sclerosis) OR Multiple Sclerosis, Acute Fulminating) (Topic) AND (non-pharmacological OR non-pharmaceutical OR non-drug intervention OR treatment OR training OR rehabilitation OR exercise OR diet OR therapy OR bright light therapy OR physical therapy OR group therapy OR repetitive transcranial magnetic stimulation OR rTMS OR deep brain stimulation OR DBS OR cognitive behavioral therapy OR CBT OR mindfulness meditation OR Baduanjin OR Qigong OR continuous positive airway pressure OR CPAP OR Tai Chi OR acupuncture OR massage therapy OR muscle relaxation OR yoga OR dance OR musicotherapy OR behavioral cognitive therapy OR psychotherapy OR assistive technique OR virtual reality)(Topic) AND (dyssomnias OR insomnia OR agrypnia OR sleep* OR sleep disorders OR sleepiness OR sleep quality OR sleep parasomnias）(Topic) AND（Randomized Controlled Trial OR controlled clinical trial OR RCT OR placebo）(Topic) |
| Cochrane Library | ‘Multiple Sclerosis’ OR ‘Disseminated Sclerosis’ OR ‘Sclerosis, Disseminated’ OR ‘MS’ in Title Abstract Keyword AND‘non-pharmacological’ OR ‘non-pharmaceutical’ OR ‘non-drug intervention’ OR ‘treatment’ OR ‘training’ OR ‘rehabilitation’ OR ‘exercise’ OR ‘diet’ OR ‘therapy’ OR ‘bright light therapy’ OR ‘physical therapy’ OR ‘group therapy’ OR ‘repetitive transcranial magnetic stimulation’ OR ‘rTMS’ OR ‘deep brain stimulation’ OR ‘DBS’ OR ‘cognitive behavioral therapy’ OR ‘CBT’ OR ‘mindfulness meditation’ OR ‘Baduanjin’ OR ‘Qigong’ OR ‘continuous positive airway pressure’ OR ‘CPAP’ OR ‘Tai Chi’ OR ‘acupuncture’ OR ‘massage therapy’ OR ‘muscle relaxation’ OR ‘yoga’ OR ‘dance’ OR ‘musicotherapy’ OR ‘behavioral cognitive therapy’ OR ‘psychotherapy’ OR ‘assistive technique’ OR ‘virtual reality’ in Title Abstract Keyword AND ‘dyssomnias’ OR ‘insomnia’ OR ‘agrypnia’ OR ‘sleep*’ OR ‘sleep disorders’ OR ‘sleepiness’ OR ‘sleep quality’ OR ‘sleep parasomnias’ in Title Abstract Keyword AND ‘Randomized Controlled Trial’ OR ‘controlled clinical trial’ OR ‘RCT’ OR ‘placebo’ in Title Abstract Keyword |
| CINAHL | （TI (Multiple Sclerosis OR Sclerosis, Multiple OR Sclerosis, Disseminated OR Disseminated Sclerosis OR MS (Multiple Sclerosis) OR Multiple Sclerosis, Acute Fulminating) SU (Multiple Sclerosis OR Sclerosis, Multiple OR Sclerosis, Disseminated OR Disseminated Sclerosis OR MS (Multiple Sclerosis) OR Multiple Sclerosis, Acute Fulminating)OR AB (Multiple Sclerosis OR Sclerosis, Multiple OR Sclerosis, Disseminated OR Disseminated Sclerosis OR MS (Multiple Sclerosis) OR Multiple Sclerosis, Acute Fulminating)) AND（TI (non-pharmacological OR non-pharmaceutical OR non-drug intervention OR treatment OR training OR rehabilitation OR exercise OR diet OR therapy OR bright light therapy OR physical therapy OR group therapy OR repetitive transcranial magnetic stimulation OR rTMS OR deep brain stimulation OR DBS OR cognitive behavioral therapy OR CBT OR mindfulness meditation OR Baduanjin OR Qigong OR continuous positive airway pressure OR CPAP OR Tai Chi OR acupuncture OR massage therapy OR muscle relaxation OR yoga OR dance OR musicotherapy OR behavioral cognitive therapy OR psychotherapy OR assistive technique OR virtual reality) OR SU (non-pharmacological OR non-pharmaceutical OR non-drug intervention OR treatment OR training OR rehabilitation OR exercise OR diet OR therapy OR bright light therapy OR physical therapy OR group therapy OR repetitive transcranial magnetic stimulation OR rTMS OR deep brain stimulation OR DBS OR cognitive behavioral therapy OR CBT OR mindfulness meditation OR Baduanjin OR Qigong OR continuous positive airway pressure OR CPAP OR Tai Chi OR acupuncture OR massage therapy OR muscle relaxation OR yoga OR dance OR musicotherapy OR behavioral cognitive therapy OR psychotherapy OR assistive technique OR virtual reality) OR AB (non-pharmacological OR non-pharmaceutical OR non-drug intervention OR treatment OR training OR rehabilitation OR exercise OR diet OR therapy OR bright light therapy OR physical therapy OR group therapy OR repetitive transcranial magnetic stimulation OR rTMS OR deep brain stimulation OR DBS OR cognitive behavioral therapy OR CBT OR mindfulness meditation OR Baduanjin OR Qigong OR continuous positive airway pressure OR CPAP OR Tai Chi OR acupuncture OR massage therapy OR muscle relaxation OR yoga OR dance OR musicotherapy OR behavioral cognitive therapy OR psychotherapy OR assistive technique OR virtual reality) ) AND（TI (dyssomnias OR insomnia OR agrypnia OR sleep* OR sleep disorders OR sleepiness OR sleep quality OR sleep parasomnias）OR SU (dyssomnias OR insomnia OR agrypnia OR sleep* OR sleep disorders OR sleepiness OR sleep quality OR sleep parasomnias）OR AB (dyssomnias OR insomnia OR agrypnia OR sleep* OR sleep disorders OR sleepiness OR sleep quality OR sleep parasomnias)) AND (TI (Randomized controlled trial* OR RCT OR RCTs) OR SU (Randomized controlled trial* OR RCT OR RCTs) OR AB (Randomized controlled trial* OR RCT OR RCTs )) |
| CNKI | Topic: (Multiple Sclerosis) AND Topic: (Sleep+Sleep Disorders+Insomnia+Sleep Quality+Sleepiness) AND Topic: (Randomized+Randomized Controlled+Trial) |
| Wanfang Database | Topic: (Multiple Sclerosis) AND Topic: (Sleep or Sleep Disorders or Insomnia or Sleep Quality or Sleepiness) AND Topic: (Randomized or Randomized Controlled or Trial) |
| VIP | Title or Keywords: (Multiple Sclerosis) AND Title or Keywords: (Sleep or Sleep Disorders or Insomnia or Sleep Quality or Sleepiness) AND Title or Keywords: (Randomized or Randomized Controlled or Trial) |
