# Supplementary figures and images for: Comparative efficacy of non- pharmacological interventions on sleep quality in patients with multiple sclerosis: a systematic review and network meta-analysis
Source: PeerJ. 2026 Mar 16;14:e20900. doi: 10.7717/peerj.20900 (PMC13001664; doi:10.7717/peerj.20900)

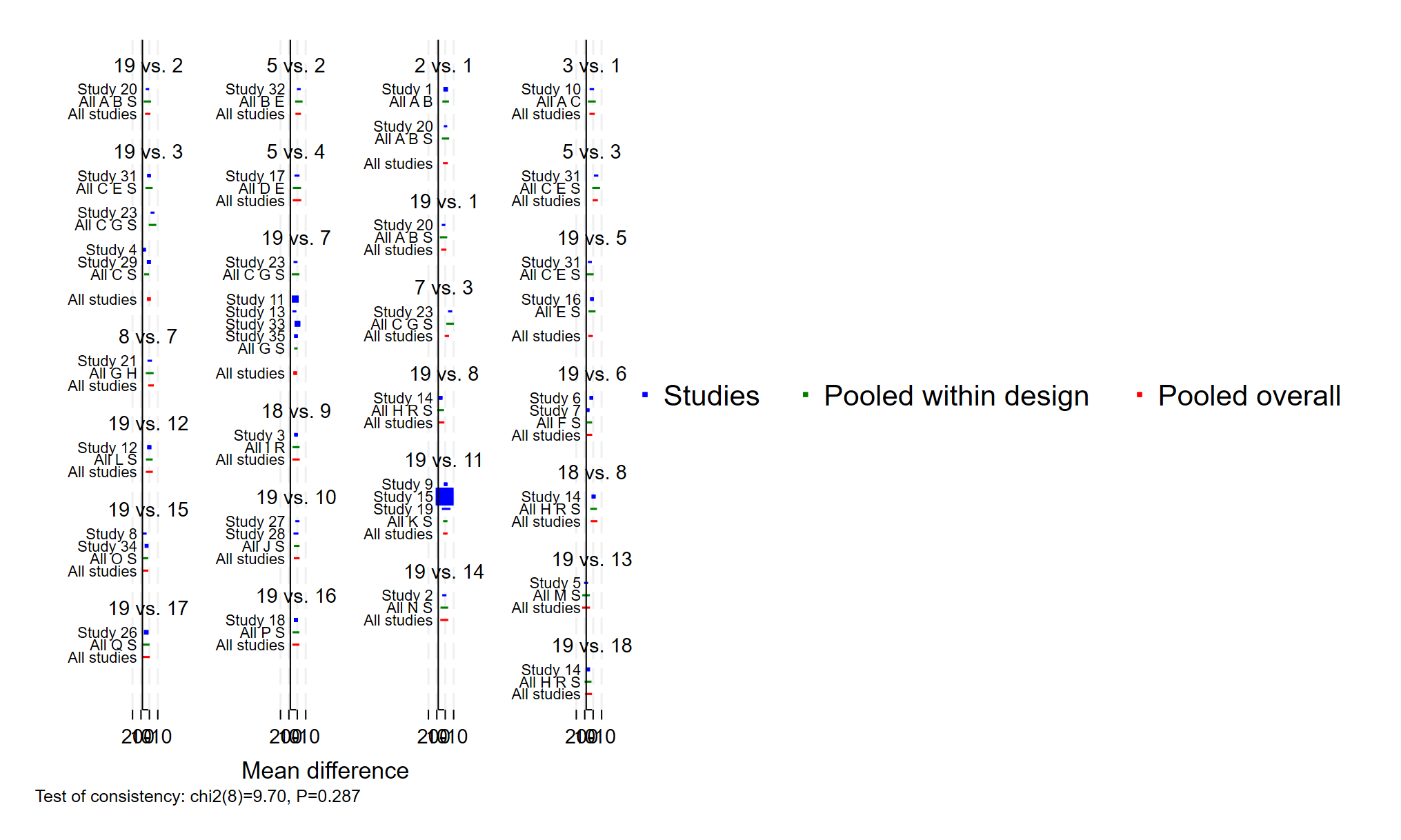

Supplement: Supplemental Information 3 [file peerj-14-20900-s003.png]

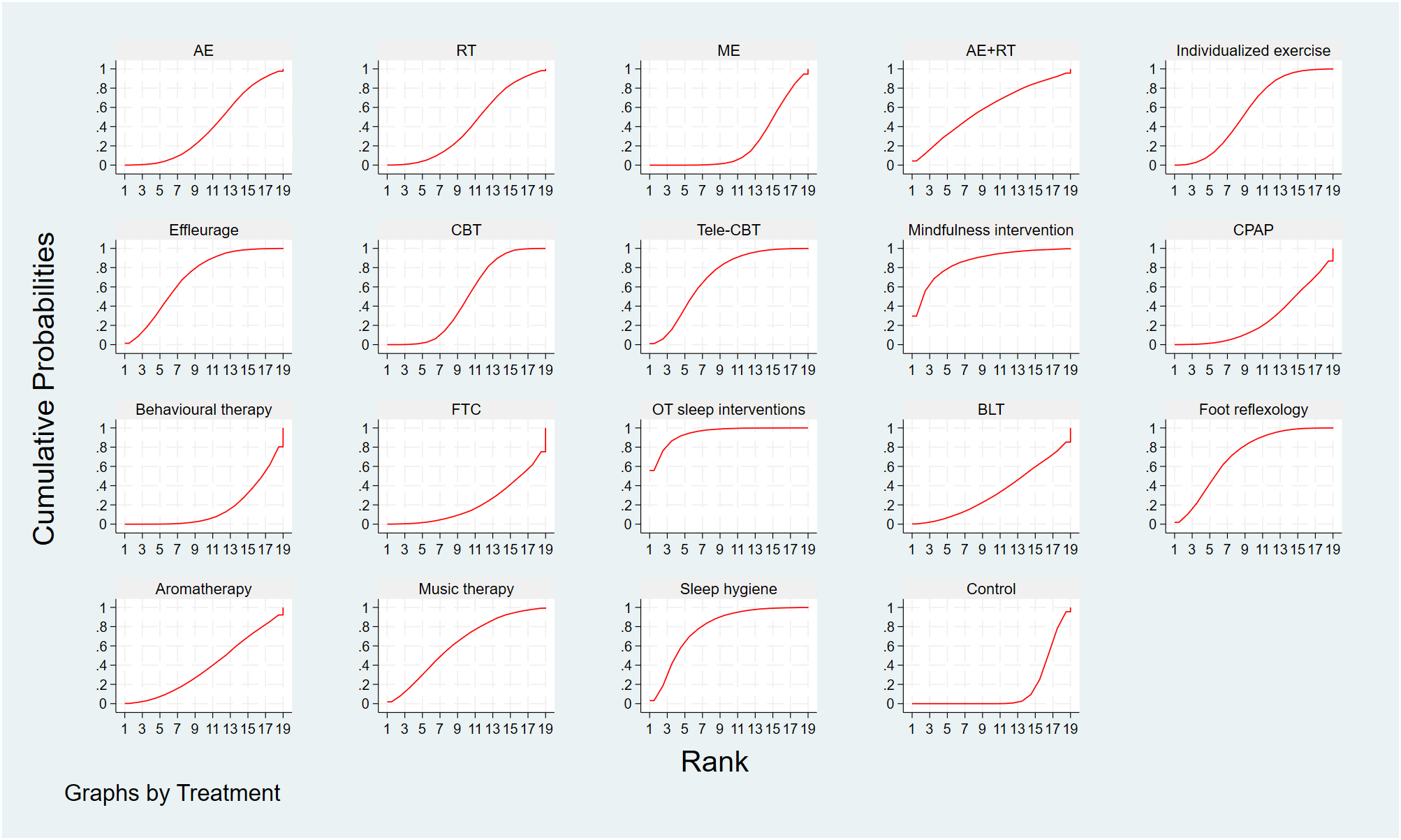

Supplement: Supplemental Information 4 [file peerj-14-20900-s004.png]

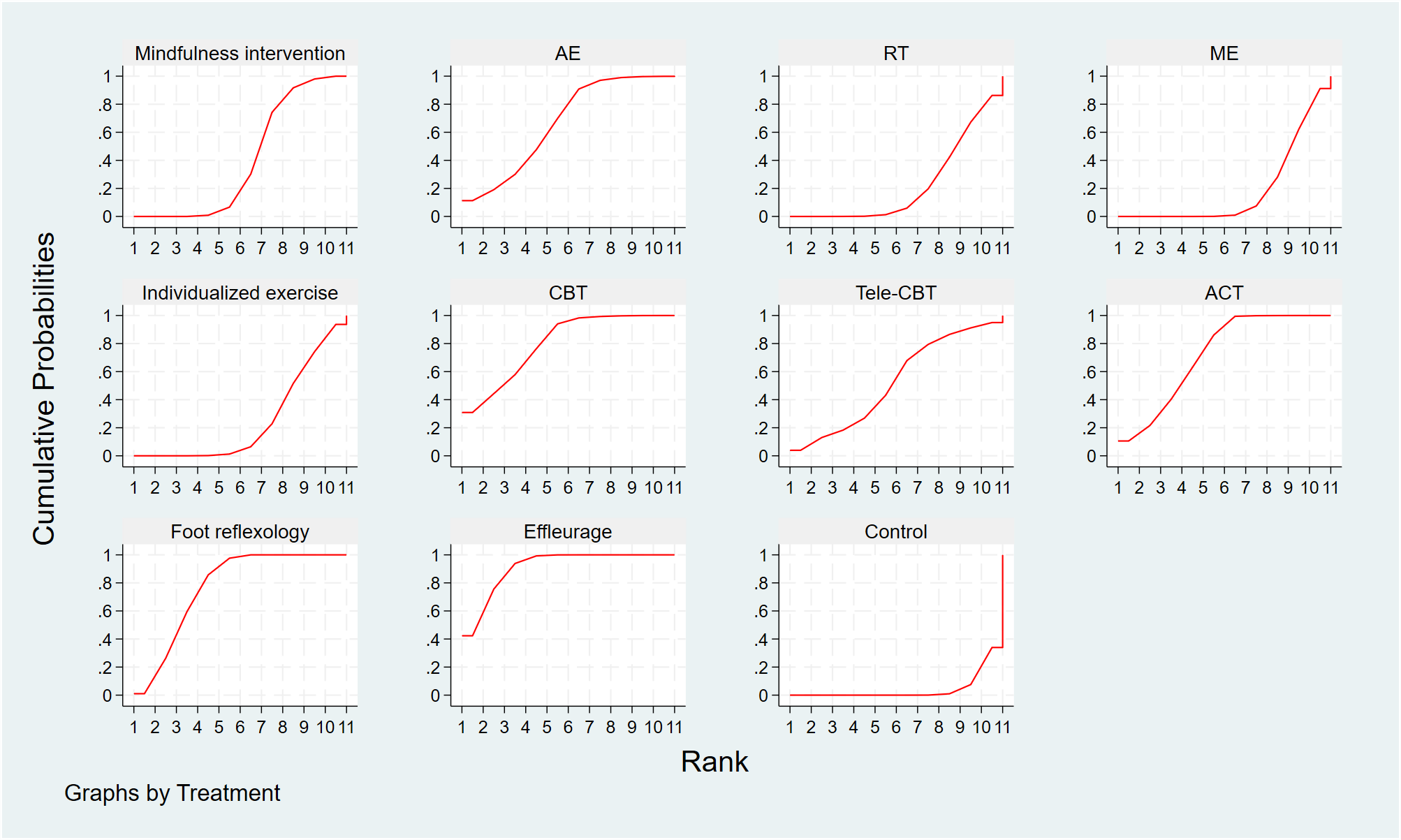

Supplement: Supplemental Information 5 [file peerj-14-20900-s005.png]
